# Post-Embolization Excision of Glomus Tympanicum: A Case Report

**DOI:** 10.7759/cureus.21414

**Published:** 2022-01-19

**Authors:** Girija Ghate, Aastha Bhatnagar, Sabreena Mukhtar

**Affiliations:** 1 Otolaryngology - Head and Neck Surgery, Dr. D.Y. Patil Medical College, Hospital & Research Centre, Pune, IND; 2 Otorhinolaryngology Head Neck Surgery, Florence Hospital, Srinagar, IND

**Keywords:** glasscock-jackson classification, pulsatile tinnitus, pre-operative embolization, glomus tympanicum, paraganglioma

## Abstract

Glomus tympanicum is a slow-growing benign tumor that can be locally destructive, spreading along the path of least resistance. Conventionally seen as soft tissue mass in the middle ear, it is difficult to distinguish glomus tympanicum from other soft tissue masses of the tympanic cavity, especially as it hides behind an intact tympanic membrane. The primary diagnostic modalities are CT scan and MRI for evaluation of the exact anatomical extent and size of the glomus tumors. Embolization following an angiographic study helps to identify the feeding arteries with subsequent blocking of the same, thus helping in the reduction of intraoperative hemorrhage. The currently available modalities of treatment are mainly surgery and radiotherapy.

Here, we report a case of a 40-year-old female who presented with unilateral deafness and tinnitus, with no co-morbidities. She showed a red bulging mass behind an intact tympanic membrane on otoscopy and otomicroscopy with mild conductive hearing loss. MRI showed an intensely enhancing lesion in the mesotympanum and hypotympanum along the cochlear promontory. A diagnosis of glomus tympanicum was made based on clinical, audiological, and radiological findings. Pre-operative embolization was carried out 48 hours before the surgery. Complete resection of the tumor was achieved by microsurgery.

## Introduction

Glomus tumors, also called nonchromaffin cell paragangliomas, arise from neural crest cells [1]. In the temporal bone, glomus tumors can either present as glomus tympanicum or glomus jugulare. Glomus tympanicum is associated with the Jacobson's nerve, a branch of the glossopharyngeal nerve. The overall incidence reported is 1 in 1.3 million cases [2]. The tumor typically arises from capillary and precapillary vessels in between epithelial cells and is thus very vascular in nature. Though benign in histology, it is a slow-growing tumor that is locally destructive, spreading along the paths of least resistance [3].

Glomus tympanicum commonly develops in the fifth to sixth decades of life. The most common presenting symptoms are conductive hearing loss and pulsatile tinnitus and are typically seen as a soft tissue mass behind the intact tympanic membrane [4]. Glomus tympanicum, especially of a large size, can cause the occurrence of vertigo, facial nerve palsy, and even hearing loss of sensory neural type [5-7].

Morbidity is associated by virtue of the anatomical location of the tumor at the skull base adjacent to the posterior cranial fossa as well as the lower cranial nerves affecting deglutition and phonation, even though the tumor is indolent and generally benign in nature. Glomus tumors spread from their site of origin along the tracts of least resistance, the most important of which are the air cells of the temporal bone. Spread occurs along several fronts simultaneously and is multidirectional. Cochleovestibular destruction is caused by ischemic necrosis [8]. Cranial nerve palsies occur in 35% of jugulo-tympanic lesions [9].

By consensus, the management of glomus tumors is surgical. Nevertheless, the surgery versus radiation therapy and stereotactic radiosurgery debate continues to rage.

## Case presentation

 A 40-year-old female patient presented to the outpatient department (OPD) with chief complaints of gradually progressive left ear hearing loss and pulsatile tinnitus for one month. She also complained of dizziness of a similar duration, which was of a spinning type not related to any postural variation. There was no history of any ear discharge, otalgia, aural fullness, or any other ear complaints. There was no history of nausea or vomiting.

**Figure 1:**
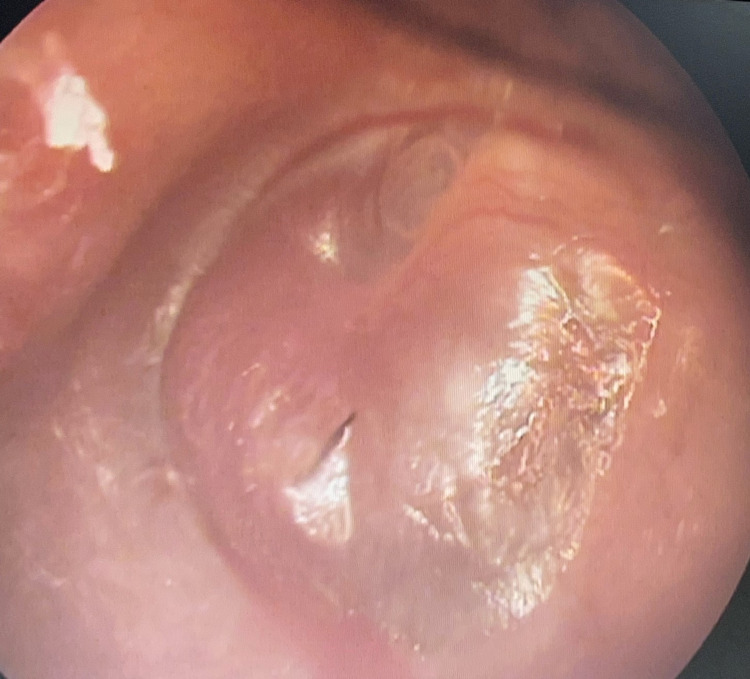
Left intact tympanic membrane showing reddish hue behind it

On otoscopic examination, the left ear showed a bright reddish hue present over an intact bulging tympanic membrane, suggestive of a vascular mass behind the tympanic membrane (Figure 1). On applying positive pressure, the mass appeared pulsatile indicating a positive Brown sign. The audiometric evaluation was suggestive of 30-decibel conductive deafness in the left ear.

**Figure 2:**
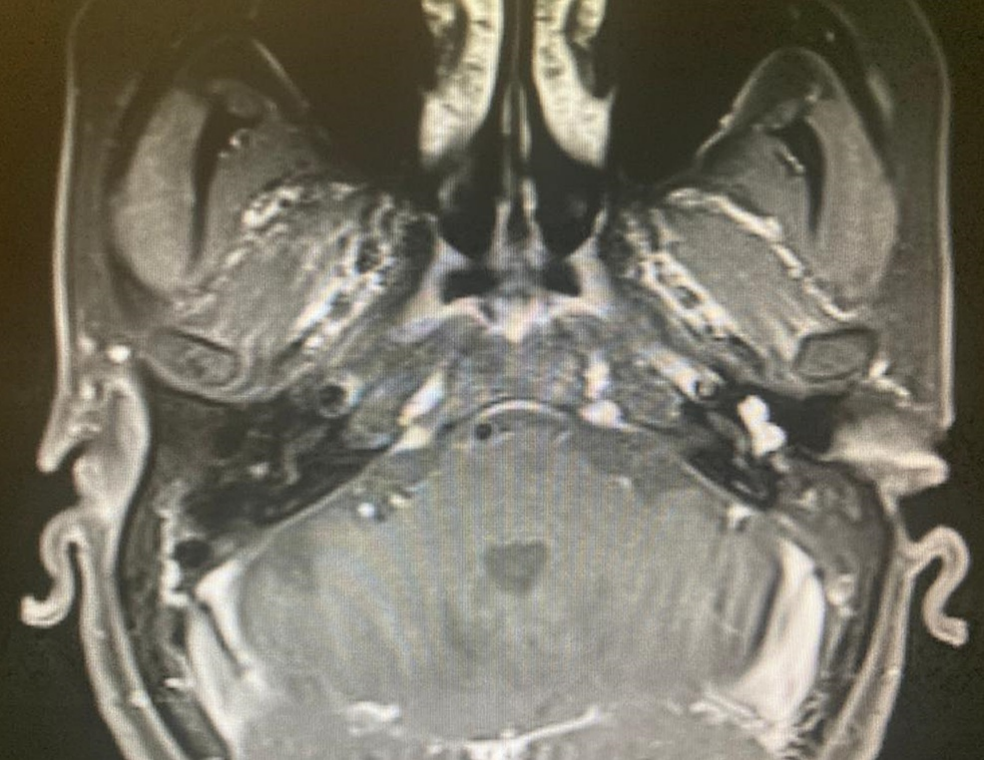
MRI of temporal bone showing the mass filling the mesotympanum

High-resolution computed tomography (HRCT) temporal bone showed a contrast-enhanced lesion occupying the mesotympanum on the left side. MRI scan of temporal bones was suggestive of a well-defined soft tissue intensity, intensely-enhancing lesion in the middle ear cavity along the cochlear promontory occupying the mesotympanum and hypotympanum with no evidence of extension into the aditus or left mastoid antrum or external auditory canal (EAC) (Figure 2). This was suggestive of Glomus Tympanicum - type II by Glasscock-Jackson classification.

**Figure 3:**
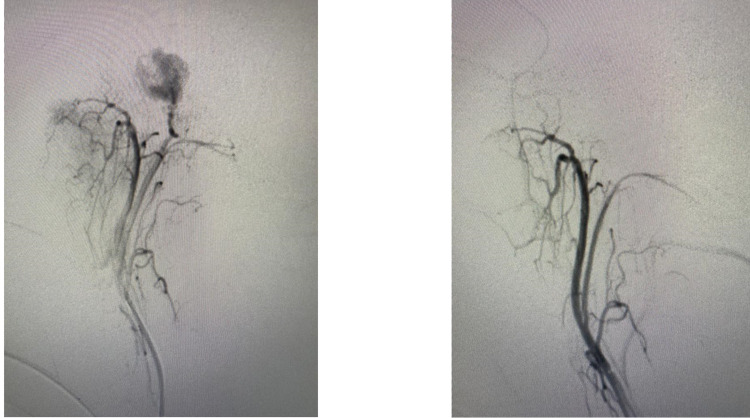
Angiography: pre-embolization (left) and post-embolization (right)

The patient was subjected to preoperative prophylactic tumor embolization after doing digital subtraction angiography (DSA), which showed the hyper-vascular tumor being supplied by the inferior tympanic branch of ascending pharyngeal artery, petrous branch of the middle meningeal artery, and stylomastoid branch of the occipital artery. The post-procedure angiogram showed a significant reduction in tumor blush with a small residual supply from the petrous branch of the middle meningeal artery and the stylomastoid branch of the occipital artery (Figure 3).

**Figure 4:**
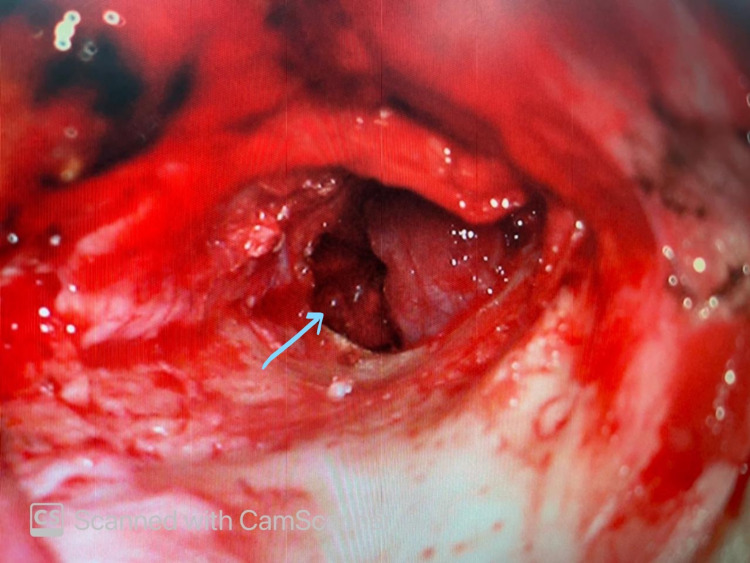
Tumor visualized in the middle ear after elevating tympanomeatal flap

**Figure 5:**
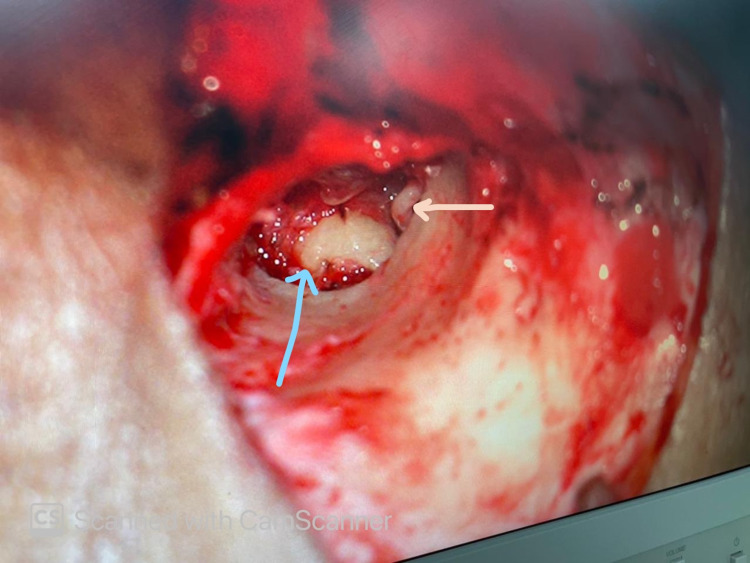
Tumor dissected off and excised exposing the promontory (blue arrow) and incudostapedial joint (pink arrow)

Post embolization, the patient was then taken for tumor excision by the post-auricular approach. After posterior meatotomy, the tympanomeatal flap was raised to inspect the tumor in the middle ear. The tumor was found to be adherent to the mucosal layer of the tympanic membrane, ossicles, and the promontory occupying mesotympanum and hypotympanum (Figure 4). Complete resection was achieved by carefully dissecting the tumor off the promontory, the handle of malleus, and the mucosal surface of the tympanic membrane (Figure 5). Minimal hemorrhage was encountered during surgery.

Histopathological examination showed well-circumscribed and well-capsulated tumors composed of vascular spaces lined by endothelial cells. Focal areas showed cells arranged in zellballen pattern or nested pattern. Immunohistochemistry was positive for synaptophysin, chromogranin, and CD 34 in blood vessels. This favored a diagnosis of Glomus tympanicum.

Postoperatively, the audiometric evaluation of the patient showed closure of the air-bone gap and, clinically, the patient improved significantly with no complaints of tinnitus and significant hearing loss anymore.

## Discussion

Arising from the neuroectodermal tissue, paragangliomas are slowly progressive, benign tumors. There are two groups of paragangliomas that are seen to occur in the head and neck region: cervical paragangliomas and temporal bone (jugulo-tympanic) paragangliomas. The jugulo-tympanic type of paragangliomas comprises glomus tympanicum and glomus jugulare while the cervical group consists of carotid body and glomus vagale tumors [1]. The incidence of glomus tympanicum tumors is more common than glomus tumors around the jugular vein, thus becoming one of the most common neoplasm of the middle ear and the second most common neoplasm of the temporal bone [1]. The Glasscock-Jackson system [8] is used to classify the tumor. Intracranial extension is expressed as a superscript.

Guild, in 1941 described “glomic tissue” in temporal bone as a vascular tissue in the dome of the jugular bulb and the promontory of the middle ear for the first time [10]. Subsequently, in 1945, a “carotid body tumor” of the middle ear and mastoid was reported by Rosenwasser as a glomus tumor [11]. Later, the term glomus tympanicum was described by Guilford and Alford as those paragangliomas that are limited to the middle ear cavity [10].

The jugulo-tympanic paraganglions are spherical, lobulated structures measuring 0.1-1.5 mm in diameter [12]. The inferior tympanic branch of the ascending pharyngeal artery commonly supplies these neoplasms. They are found to be associated with Jacobson's (tympanic branch of the glossopharyngeal nerve) and Arnold's nerves (auricular branch of vagus nerve) [3].

The biochemical capacity of the glomus tumors is indeed rich. Its potential to produce neuroendocrine secretory products permits antici­pation of a variable clinical symptomatology and those tumors that secrete sufficient quantities are known as "functional" tumors or "secretors." Norepinephrine levels elevated three to five times normal generally produce the symp­toms and signs of catecholamine secretion, such as headaches, excessive perspiration, palpitations, pallor, and nausea [13].

Perioperative management is essential to safeguard against grave consequences of catecholamine overload on anes­thesia induction or intraoperatively on tumor manipulation. Modern protocols for pharmacologic blockade employed for a pheochromocytoma are used [6]. Alpha- and beta-blockade begin­ning two weeks preoperatively has been abandoned. Paraneoplastic syndromes associated with other neuro­ hormones (anemia, gastrointestinal symptoms, etc.) must be sought and identified. 

The clinical features of glomus tumors serve to alert the physician to a disorder of the ear, temporal bone and jugular fossa. The patient with glomus tumor usually complains of pulsatile tinnitus and/or hearing loss. Conductive hearing loss occurs as a result of the tumor growth into the mesotympanum and the laby­rinthine invasion determines the degree of the sensorineural component depending on the level of extent. Tympanic membrane erosion and bleeding are late symptoms [3]. Cranial neuropathy suggests a more extensive pro­cess. As lower cranial nerves are affected, dysphagia, loss of airway protection, and shoul­der, tongue, and voice weakness occur. Facial paralysis is usually a late sign indicating a poor prognosis. The signs and symptoms of "functioning" glomus tumors must be sought and differentiated from pheochromocytoma.

A mesotympanic vascular mass is characteristic but rarely may be absent. Superior mesotympanic masses can occur in glomus tumors but are rare and diagnostically confusing. Margins visible 360 degrees about the circumference of a mesotympanic mass per­mit the diagnosis of a tympanicum lesion [11]. Without this physical finding, differentiation of a glomus tympanicum tumor from glomus jugulare tumor is insecure and impossible without imaging.

In view of its location, a successful resection of these tumors may become a very challenging process for the treating surgeon. This may further be complicated by the vascular nature of the tumor. As a measure of caution, an elective preoperative embolization can be planned, which will help bleeding complications [14]. Therefore, keeping in mind the benefits of interventional radiology and site and size of tumor as well patient profile, we preferred this modality of treatment for our patient. Due to the highly vascular nature of the tumor, incisional biopsy is contraindicated. Tissue diagnosis, when necessary to differentiate it from others, should be done through surgical resection and histopathological examination.

Our patient presented with a classical combination of features viz. conductive hearing loss, tinnitus and a red bilging mass behind an intact tympanic membrane. MRI showed a vascular mass occupying the middle ear completely (Glasscock-Jackson Type II tumor). Inferior tympanic branch of the ascending pharyngeal artery, petrous branch of the middle meningeal artery, and stylomastoid branch of the occipital artery. Blood supply was mainly noted from the inferior tympanic branch of the ascending pharyngeal artery, petrous branch of middle meningeal artery and stylomastoid branch of the occipital artery. After appropriate embolization, a residual blood supply from the middle meningeal artery was noted. The patient underwent surgical excision during which the tumor was found exclusively in the middle ear, over the promontory, around the handle of malleus and near the incudostapedial joint. This coincided with the radiological finding of the Glasscock-Jackson type II tumor. Excision was in toto with tumor size being 0.8 cm approximately. 

## Conclusions

As in our case, the treating physician should also consider the differential diagnosis of a glomus tumor so as to prevent any disastrous complication such as an intraoperative hemorrhage. Physicians should be open to modalities such as preoperative embolization as it has shown promising prevention of excessive intraoperative hemorrhage, as was done in our case. Preoperative planning and approach should also be planned with due consideration of all the risks involved.

Therefore, in any patient presenting with reduced hearing, vertigo, and tinnitus, a careful otological and otomicroscopic examination is absolutely necessary during a clinical examination by all treating physicians. Also, the role of imaging and interventional radiology cannot be stressed more for better diagnostic and therapeutic management.
